# Acute anabolic response to β-hydroxy-β-methylbutyrate (HMB)-free acid supplementation following heavy resistance exercise

**DOI:** 10.1186/1550-2783-11-S1-P16

**Published:** 2014-12-01

**Authors:** JR Townsend, JR Stout, JR Hoffman, AM Gonzalez, AR Jajtner, AJ Wells, CH Boone, KS Beyer, GT Mangine, EH Robinson, GJ Pruna, JD Bohner, MS Fragala, DH Fukuda

**Affiliations:** 1 University of Central Florida, Orlando, FL, USA

## Background

β-hydroxy-β-methylbutyrate (HMB), a metabolite of the amino acid leucine, has been shown to promote strength and lean muscle mass when supplemented in conjunction with resistance training. Recently, a new free-acid form of HMB has been shown to reach higher plasma concentrations in a shorter amount of time compared to the calcium-salt form. This higher bioavailability may rationalize acute supplementation with HMB-FA as a means to enhance the anabolic response resulting from heavy resistance training. The purpose of this study was to examine the effect of acute β-hydroxy-β-methylbutyrate-free acid (HMB-FA) on circulating concentrations of anabolic hormones following a heavy resistance exercise protocol.

## Methods

Twenty resistance-trained men (22.8 ± 2.5 yrs, 177.6 ± 6.6cm, 83.4 ± 9.8kg) volunteered to participate in this study and were randomized into two groups [HMB-FA and placebo (PL)] and performed an acute, heavy resistance exercise protocol (four sets of up to 10 repetitions of the squat, dead lift, and split squat exercises). Supplementation included 1 g of HMB-FA or PL consumed 30min prior to exercise. Blood was sampled before (PRE), immediately post (IP), and 30 min post-exercise (30P). Circulating levels of testosterone (TEST), growth hormone (GH) and Insulin (INS) were assayed. A 2 x3 repeated measures ANOVA was used to analyze the data. Consent to publish the results was obtained from all participants.

## Results

The resistance exercise protocol produced a significant time effect for an elevation in TEST (p<0.01), GH (p<0.01) and INS (p = 0.05) at IP with GH (p<0.01) and INS (p<0.01) remaining elevated at 30P. A group by time interaction was observed (p=0.05) with plasma GH elevated in HMB-FA compared to PRE values (p < 0.01) at IP and 30P. There were no differences at any other time point with TEST or INS concentrations.

## Conclusion

These data indicate that HMB-FA supplementation may augment and prolong the growth hormone elevation associated with heavy resistance exercise.

